# Do Public Pensions Improve Mental Wellbeing? Evidence from the New Rural Society Pension Insurance Program

**DOI:** 10.3390/ijerph18052391

**Published:** 2021-03-01

**Authors:** Fang Wang, Haitao Zheng

**Affiliations:** School of Economics and Management, Beihang University, Beijing 100191, China; wangf_master@126.com

**Keywords:** pension income, mental wellbeing, depression, regional differences, rural elderly

## Abstract

The causal effect of public pensions on the mental wellbeing of the elderly in lower and middle-income countries deserves further investigation. This paper first constructed a theoretical framework for the impact of New Rural Society Pension Insurance pensions in China on the mental wellbeing of the rural elderly, and described potential channels through which pension income may affect mental wellbeing. We then used the fixed effect model and the instrument variable approach to estimate the casual effects of pension income on the mental wellbeing of the rural elderly. The results reveal that pension income improves mental wellbeing by relieving depression of the rural elderly; however, the beneficial effects of pension income are very limited. Pension income has no beneficial effects on the mental health of the rural elderly in the east region, whereas it slightly relieves depression of those in the middle and west regions. We also found that pension income produces small improvements in the mental health of older females, elderly persons living independently, and those with relatively poor economic conditions.

## 1. Introduction

The unprecedented population aging brings one of the biggest challenges for lower and middle-income countries (LMICs) to ensure the wellbeing of their growing old population. According to the statistics of the United Nations, the world’s old population was over 1.04 billion in 2020 [1], will reach 1.97 billion by 2050 [2], and more than 80% of them will be living in LMICs [3]. As one of the most important components of social security systems, social pension or government transfer programs were recently initiated for the uncovered population in many LMICs [4]. As other LMICs, China has been undergoing a rapid population aging. The proportion of population aged 60 years and above increased from 10.33% in 2000 to 13.26% in 2010 according to the Sixth National Population Census in China [5], increased to 18.10% in 2019 according to the National Bureau of statistics [6], and this trend is projected to rise to 32.8% by 2050 [7]. Moreover, population aging in rural areas is more serious than in cities and towns. Thus, China has been implementing the New Rural Society Pension Insurance (NRSPI) program since 2009, covering all of the 2853 county-level administrative regions and 460 million rural residents in 2012, the largest pension program in the world.

The global prevalence of mental disorders has been increasing over the past decades, and mental disorders have been recognized as a leading cause of disability and death [8] and a major contributor to non-fatal health loss worldwide [9]. The LMIC population has more than twice the rate of mental disorders compared with their U.S. counterparts [10]; however, investment in mental illness prevention and treatment remains low in LMICs, and between 76% and 85% of people with severe mental disorders receive no treatment [11]. As the most common and serious mental disorder, the incidence of depression increased by 18.4% between 2005 and 2015, depressive disorders led to more than 50 million Years Lived Disability (YLD) in 2015, accounting for 7.5% of all YLDs, and more than 80% of this non-fatal disease burden occurred in LMICs [12]. Moreover, depression occurs at a higher level among elderly persons, and prevalence of depression at older ages has been increasing as a result of global population aging [12,13].

It is of great significance that LMICs improve the mental wellbeing of their growing old population. There is considerable evidence that financial difficulty is the major barrier for the old population in LMICs to access suitable health services [14,15]. While providing the elderly with income security, social pension or government transfer programs may have important implications for the health and wellbeing of the elderly [4]. Although, a large number of studies have investigated the causal effect of public pensions to economic wellbeing of older adults in LMICs, there is currently a dearth of research on health consequences, especially mental health consequences associated with public pensions. This paper provides new causal evidence of public pensions on mental wellbeing in the LMIC context. Further understanding of the causal effect of public pensions on mental wellbeing, measured by depression score and depressive symptoms, is of significant importance for LMICs in order that they can develop more effective pension programs as well as mental health prevention and treatment policies.

Early studies based on developed countries found a positive correlation between income or wealth and health [16,17], which exists for mental as well as physical health, but the degree to which this relationship was causal remains a crucial open question that deserved further investigation [18,19]. Emerging literature has attempted to estimate the causal effects of income on mental health by using quasi-experimental study design or panel data models. A study using an instrumental variable approach showed significant improvement in mental health for United States adults due to increased income [20]; however, the validity of instrument variables was questioned [21,22]. Recently, studies using British and Swedish lottery winnings as an exogenous source of income variation [23,24,25], migration to higher living standards in New Zealand [26], changes in child benefits in Canada [27] all found positive causal linkages between income and mental health. However, Stowasser et al. [28] failed to find compelling evidence for this positive causal linkage. Given well-functioning social security systems in developed countries [29], literature on pension-mental health gradient is scant in these countries. Golberstein [30] contributed to the literature by assessing the causal effects of Social Security income on mental health of United States older adults, and indicated improvements in depressive symptoms and lower probability of being diagnosed with mental disorders only for women.

LMICs usually provide limited social safety nets, social pensions or government transfers might be more important for mental health production of the beneficiaries in these countries. Exogenous increases in South African state old age pension income improved physical and mental health status of pensioners and other household members [31], whereas LIoyd-Sherlock and Agrawal [14] found no evidence that pension income was positively associated with mental wellbeing. A study in Mexico revealed that a social pension program significantly decreased the depression score by 12% and improved mental health of the beneficiaries [32]. Two recent studies on the NRSPI program in China both found that the NRSPI pensions significantly improved mental health of rural elderly by decreasing the depression score or depressive symptoms [13,33].

However, Cheng et al. [33] measured psychological wellbeing by total score of only six items, which has been less used in standard psychological health measures in the literature. They also did not analyze the impact of pension income on the rate of depressive symptoms, which may be more important to assess the seriousness of depression. Chen et al. [13] used cross-sectional data in 2012 to examine the effects of pension enrollment and income on mental health, which makes it difficult to control individual effects and to analyze long-term impact of pension income. In addition, Cheng et al. [33] and Chen et al. [13] mainly assessed the impact of pension income on mental health of rural elderly, but failed to describe mental health production within the context of the NRSPI pensions and to discuss regional differences of the impact of pension income.

This paper focuses on investigating the impact of the NRSPI pensions on the mental health of rural elderly and contributes to the literature in three ways. First, it helps us understand mental health production of the rural elderly within the context of the NRSPI pensions. Based on the standard static health production model [24,34,35] and the agricultural household decision-making model [36,37], this paper constructs a theoretical framework to analyze the effects of the NRSPI pensions on mental wellbeing of the rural elderly and to discuss potential channels from pension income to mental health.

Second, the current study estimates the impact of pension income on the mental health of the rural elderly by using the fixed effect model (FE) and the instrument variable approach. Due to the voluntary principle of the NRSPI participation, pension receipt may be an endogenous household decision, which may be related to unobservable household characteristics [38]. The fixed effect model with instrument variable (FE-IV) can control individual effects and the endogeneity of pension income simultaneously. In addition, we measured mental health by total depression score and whether the respondents had depressive symptoms or high depressive symptoms based on the 10-question version of the Center for Epidemiologic Study depression battery.

Third, this study contributes to the literature by first investigating regional differences in the impact of the NRSPI pensions. Regional difference is an important factor that should be considered in the assessment of the impact of the NRSPI program [39], and pension income may generate a larger impact in regions that are lagging behind in economic growth [40]. According to the regulations, central government finance half the basic pension for the east region, whereas the whole costs for the relatively poor middle and west regions. Thus, we analyzed regional differences of the impact of pension income on the mental health of the rural elderly, and found that pension income does not decrease depression of elderly persons in the east region, whereas it slightly relieves depression of those in the middle and west regions.

Furthermore, we investigated the heterogeneous effects of pension income to determine how elderly persons from different gender groups, household income brackets, and living arrangements, behave with regard to their mental wellbeing, and found that older females, elderly persons living independently, and those among the lower income group benefit from the NRSPI pensions.

The rest of this paper is organized as follows. Section 2 introduces the Chinese NRSPI program. Section 3 provides sample data and their statistical description. Section 4 develops theoretical framework and econometric models. Section 5 provides empirical results and analyses. Section 6 summaries findings and discusses policy implications.

## 2. Chinese NRSPI Program

China has two main parallel public pension systems, a pension system for urban employees and a pension system for urban and rural residents. This study focuses on the pension system for rural residents, the NRSPI program. The government released guidelines for implementation of the New Rural Society Pension Insurance program in September 2009, and carried it out in 10% of counties across the country [41]. By 31 August 2012, all of the 2853 county-level administrative regions had implemented the program, achieving full geographic coverage. By the end of 2012, the number of rural residents enrolled had reached to 460 million, the highest in the world. To build a social security system covering urban and rural residents, State Council decided to merge the NRSPI system and the Urban Residents’ Pension system into a unified system, the Urban and Rural Residents’ Pension Insurance, in February 2014 [42].

The NRSPI system is a partially funded system, that is, the funds of the program consist of individual payments, together with collective and government subsidies. Standards of individual payment are initially set at CNY100–500 every year, payment standards of CNY600–1000, 1500 or 2000 are added in a merged system, and local government can determine additional payment grades according to actual situations. Local residents aged 16 years and above, excluding students, who do not enroll in basic old age insurance for urban employees, can participate and contribute to the program according to the aforementioned standards. If possible, a village collective should provide subsidies to the participants. Local government should provide no less than CNY30 for the participants every year in the initial system, and no less than CNY60 in the current system.

When participants are 60-years-old, they can begin to receive NRPSI pensions, which comprise basic pensions and income from personal accounts. Basic pensions are initially set at CNY55 per person per month, raised to CNY70 in 2014, and further increased to CNY1056 per year in 2018 [43]. Basic pensions are entirely financed by central government in the middle and west regions, 50% are financed by central government and the remaining 50% are financed by local government in the east region. If rural residents have reached 60 when the program is implemented, they are eligible to receive the non-contributory basic pensions, but their eligible children must enroll in the program. This feature is typical of the program and different from public pension systems of other nations. For participants aged 16 to 59, they can receive basic pensions and pension benefits from personal accounts at 60. Monthly pension benefits from personal accounts equals the total amount of personal accounts divided by the divisor coefficient 139, which is based on the average life expectancy of the population, retirement age, and interest rate.

## 3. Data and Stylized Facts

The NRSPI program was implemented in 2009, the Urban Residents’ Pension program was implemented in 2011, and the NRSPI program and the Urban Residents’ Pension program were further merged in 2014. These two pension systems were still operating in parallel before 2014; thus, we used the 2011 and 2013 wave of China Health and Retirement Longitudinal Study (CHARLS), conducted by the National Development Institute of Peking University [44]. Based on the Health and Retirement Study and related aging surveys, such as the English Longitudinal Study of Aging and the Survey of Health, Aging and Retirement in Europe, CHARLS adopted a multi-stage stratified sampling method to collect a high quality nationally representative sample of Chinese residents aged 45 years and over from 450 communities (villages) in 150 county-level units of 28 provinces, excluding Hainan, Tibet, and Ningxia. CHARLS provides extensive information at the individual and the household level, including demographics, family structure, health status, health care and insurance, work, retirement, pension, income, consumption and so forth, which provided an ample data source for this research.

According to the aim of this paper, we restricted the study sample to the rural elderly, because only these respondents are eligible to receive pensions. We excluded respondents who had enrolled in pension programs other than the NRSPI program because participation in other programs would also affect the mental health of the respondents, and whether they have enrolled in other programs is also related to whether they decide to participate in the NRSPI program—covering these observations could have led to biased estimation. We connected the household data with the individual data by making the main respondents the householders and deleted observations with missing information. Our study sample was a panel composed of 1362 rural households who were surveyed in both waves.

The CHARLS adopts a 10-item CES-D measure for mental health conditions over the past week, including eight questions on negative feelings or behaviors and two on positive feelings, reported in Table S1. Respondents were asked to indicate how often they had those feelings or behaviors from four options, that is, “rarely or none of the time (less than 1 day)”, “some or a little of the time (1–2 days)”, “occasionally or a moderate amount of the time (3–4 days)”, “most or all of the time (5–7 days)”. Numbers from 0 to 3 were assigned to the four options for negative questions and scoring was reversed for positive questions. CES-D score, sum of all the responses, ranged from 0 to 30, and a higher score indicates lower mental health. Moreover, an individual was diagnosed with depressive symptoms if his or her CES-D score is 8 or greater [45,46], and with high depressive symptoms 10 and above [35,47].

Table 1 reports the CES-D score and depressive symptoms for pensioners and non-pensioners based on pooled data. According to Table 1, the number of pensioners is 1103, approximately accounting for 40.5% of the study sample. Pensioners have a lower mean CES-D score than non-pensioners, and are less likely to suffer from depressive symptoms or high depressive symptoms.

Table 2 reports the CES-D score and depressive symptoms of pensioners and non-pensioners by region based on pooled data. The number of pensioners is 217 in the east region, and 835 in the middle and west regions, approximately accounting for 31.2% and 43.9% of the sample respectively. Pensioners have a lower mean CES-D score and are less likely to have depressive symptoms or high depressive symptoms than non-pensioners both in the east region and in the middle and west regions, but the differences between pensioners and non-pensioners is more obvious in the middle and west regions. Data in Table 2 also reflects that prevalence of depression is unevenly distributed, and those who live in the middle and west regions are more likely to be depressed.

Table 3 reports statistical characteristics of independent variables based on pooled data. Pension is the natural logarithm of the NRSPI pension income received; Age the ages of the main respondents; Married the marital status, married equals 1, and otherwise 0; Family the number of household members; Children the number of children alive; Insurance whether the respondents have health insurance, *yes* equals 1, and *no* 0; Chronic the number of diagnosed chronic diseases; Land the cultivated land area operated; Liquidity natural logarithm of financial assets, including cash, deposit, government bonds, stock, and funds. In comparison with non-pensioners, pensioners are more likely to be of an older age, to have health insurance, and to report better health conditions. Pensioners have less family members, more living children, and reported more cultivated land operated and financial assets than non-pensioners.

## 4. Theoretical Framework and Econometric Models

### 4.1. Theoretical Framework

Following the literature [24,34,35,36,37], we assume a utility function $U\left(\cdot \right)$, defined over consumption C, health H, and leisure L. An individual’s health, including mental health, is produced by his or her consumption in health inputs M, individual psychosocial stress P, initial physical and mental health status $H_{0}$ and socioeconomic characteristics X. In addition to health production function, maximization of $U\left(\cdot \right)$ is also subjected to a budget constraint and a time constraint:

(1) $$MaxU=U\left(C,H,L\right)$$

(2) $$s.t.\;H=H\left(M,P;H_{0},X\right)$$

(3) $$P_{C}C+P_{M}M=Y+TC+NRP+OT$$

(4) $$T=T_{F}+T_{IF}+T_{C}+T_{H}+L$$

where Y denotes household income, TC net transfers from children; NRP pension income from the NRSP program; OT other transfers; T total stock of time; $T_{F}$ time allocated in formal work, $T_{IF}$ time allocated in informal work, $T_{C}$ time allocated in consuming other goods; $T_{H}$ time allocated in health production.

We do not solve optimization of the theoretical framework, but focus on explaining potential channels through which pension income affects mental health. According to the theoretical framework, one more direct channel goes through individual psychosocial stress [30]. Positive income shocks may improve mental health by reducing financial stress, increasing self-esteem and sense of control [13,48,49], and improving the perceived economic situation [33,50,51].

Another obvious channel goes through health inputs. Only 8% of Chinese people with mental illness receive treatment [52], due to widespread stigma regarding mental illness [53], poor mental health literacy [54], and limited ability to treat mental illness [55]. Thus, the health investment channel is mainly indirect, through health care service utilization as well as adherence to recommended treatment plans [40,56]. However, this is not necessarily associated with improved mental health [14].

The NRSPI pensions may lead to changes in food consumption and risky behaviors by relaxing budget constraint according to Equation (3). Poor nutrition is associated with adverse psychological health outcomes [57], a positive shock to income promotes nutrition intake [58], and thus relieves depression [33], whereas increased unhealthy behaviors, such as smoking and drinking are detrimental to health [24].

The NRSPI pensions may have an impact on labor supply by reducing the probability of doing physically demanding work [59], the time spent on caring for grandchildren [56], and doing arduous household chores [60], thus leading to a change in leisure, and participation in social and leisure activities having beneficial effects on mental wellbeing [61]. However, Ning et al. [62] found that the labor supply decision and work time of the elderly were not affected by the NRSPI program.

More than 70% of the participants under 60 contribute CNY100 per year, the pensions they begin to receive at 60 mainly come from basic pensions; the participants aged 60 years and above when the program is carried out can directly receive basic pensions. According to a government report, the average amount of basic pension was CNY78.6 per person per month in March 2013, approximately accounting for 17% of the average per capita living expenses among Chinese rural households in 2012, less than one-half of the amount representing the rural poverty line.

The NRSPI program provides a prevalent but modest income security, such a modest pension program may improve the mental wellbeing of the rural elderly in a short period of time, and the beneficial effects over a long period of time may be very limited. In addition, these beneficial effects may be muted by a likely crowding-out effect on transfers from adult children [56,63]. Thus, whether the NRSPI pensions help to improve the mental wellbeing of the rural elderly requires further verification.

### 4.2. Econometric Models

This paper focuses on assessing the impact of the NRSPI pensions on the mental health of the rural elderly by using panel data models, the dependent variable is the CES- D score, depressive symptoms or high depressive symptoms, and the independent variable of most attention is the NRSPI pension income received. Following Cheng et al. [33], our model is specified as

(5) $$MH_{it}=\beta_{0}+\beta_{1}NRP_{it}+\beta_{2}X_{it}+\theta_{i}+\varepsilon_{it}$$

where $MH_{it}$ denotes the CES-D score of the respondent i in the year t, whether the respondent suffers from depressive symptoms or high depressive symptoms; $NRP_{it}$ the natural logarithm of pension income from the NRSPI program; $\beta_{1}$ the impact of a 100- percent increase in pension income on mental health outcomes; $X_{it}$ a set of control variables, including age, quadratic age, marital status, number of family members, number of children alive, health insurance status, number of diagnosed chronic diseases, cultivated land area operated and natural logarithm of financial assets; $\beta_{2}$ the impact of control variables on mental health outcomes; $\theta_{i}$ the individual effect at the household level, and $\varepsilon_{it}$ the error term. There are correlations among error terms of the respondents in the same village, so the standard error estimated is clustered at the village level.

According to the regulations, participants aged 60 years and above are eligible to receive pensions. For local residents under 60 years, they voluntarily enroll in the program and begin to receive pension benefits at 60; for those aged 60 years and above, they can directly receive basic pensions as long as their eligible children enroll in the program. Thus, pension receipt may be related to unobservable characteristics, these characteristics may also affect mental wellbeing, and lead to an endogenous problem. In the fixed effect model, $X_{it}$ is allowed to be correlated with $\theta_{\;i}$, but is not allowed to be correlated with $\varepsilon_{it}$. In the random effect model, $\theta_{\;i}$ is purely random and uncorrelated with $X_{it}$. Thus, the fixed effect model can control unobservable characteristics which are time-invariant.

The fixed effect model can control unobservable and time-invariant household characteristics, but cannot totally control unobservable characteristics which vary over time. To solve this problem, according to the time when the NRSPI program is implemented in different areas, we define whether sample areas have carried out the NRSPI program as a dummy variable $CNRP_{it}$, and take it as an instrument variable (IV) for $NRP_{it}$ [64]. The first-stage regression is as follows:

(6) $$NRP_{it}=\alpha_{0}+\alpha_{1}CNRP_{it}+a_{2}X_{it}+\theta_{i}+\eta_{it}$$

where $\alpha_{1}$ measures the impact of whether the NRSPI program is implemented in sample areas on the NRSPI pensions received.

The specification of second-stage regression is the same as Equation (5). A good IV must satisfy two conditions: IV must be uncorrelated with $\varepsilon_{it}$ and partially correlated with $NRP_{it}$ once other exogenous variables have been netted out [65]. Because the time when the NRSPI program was implemented in sample areas was determined by central government, $CNRP_{it}$ is uncorrelated with the mental health of the respondents. Because the NRSPI implementation determines whether the respondents have opportunities to obtain pension income, $CNRP_{it}$ fulfills the aforementioned two conditions. Moreover, we report the first-stage regression results, and F test statistics of our IV. The F test statistic that is high enough (more than 10) indicates that $T_{it}$ is a strong IV [66].

## 5. Results and Analyses

### 5.1. Impact of the NRSPI Pensions on Mental Health Outcomes

Based on the theoretical framework, this paper constructs fixed effect models to estimate the impact of the NRSPI pensions on mental health outcomes of the rural elderly. The results are shown in Table 4. To test the robustness, we report the results of fixed effect regression with and without instrument variable, simultaneously.

According to the results of the second-stage regression, the effects of pension income on CES-D score, rates of depressive symptoms, and high depressive symptoms are all negative and insignificant in the FE regression. Controlling the endogeneity of pension income, the CES-D score is 0.207 points lower among pensioners, rates of depressive symptoms and high depressive symptoms are 1.3 and 0.1 percentage points lower respectively, and the coefficients are all insignificant, which is inconsistent from the results of Chen et al. [13] and Cheng et al. [33]. This finding is observed because pension income may generate more detectable improvements in the mental health of the rural elderly in a short period of time through the aforementioned pathways [67], whereas the beneficial effects over a long period of time are very limited.

We examine the effects on each item of CES-D to better understand potential channels from pension income to mental health. Table 5 illustrates that pension income improves nine items except “I felt everything I did was an effort”, and the coefficients of responses to “I felt fearful” and “I felt lonely” both are significant in the FE regression. Controlling the endogeneity of pension income, the results of the FE-IV regression show that pension income improves eight items except “My sleep was restless” and “I was happy”, and only the coefficient of the responses to “I felt depressed” is significant at the statistical level of 5%.

Therefore, the NRSPI pensions produce small reductions to the CES-D score and rates of depressive symptoms of the rural elderly by significantly improving their responses to “I felt depressed”. In addition, differences between the FE estimates and the FE-IV estimates indicate the importance of using the instrument variable to solve the endogeneity of pension income.

### 5.2. Regional Differences in the Impact of the NRSPI Pensions on Mental Health Outcomes

Differences exist in the implementation and financial subsidies of the NRSPI program between the east region, and the middle and west regions; thus, we divide sample areas into the east, middle, west regions according to the Division of China’s Economic Region, to analyze regional differences in the impact of the NRSPI pensions on mental health. The results of the FE-IV regression are reported in Table 6.

The results of the second-stage regression in Table 6 show that pension income has positive effects on CES-D score, and rates of depressive symptoms and high depressive symptoms in the east region, and the coefficients are all insignificant. In the middle and west regions, CES-D score, and rates of depressive symptoms and high depressive symptoms are 0.233 points, 1.9 and 0.4 percentage points lower among pensioners respectively, and the coefficients are all insignificant. Thus, the impact of the NRSPI pensions exhibits small regional differences: pension income does not decrease depression of the rural elderly in the east region, whereas it slightly relieves that of those in the middle and west regions.

This finding is explainable because the east region is economically more well-developed, rural elderly have higher income and can afford their old-age support, a small annuity income and matched government subsidies cannot produce beneficial effects on their mental wellbeing. In contrast, the middle and west regions are economically under-developed areas, the rural elderly have lower income, so pension income can make changes to their lifestyle and help reduce their financial stress while serving to improve mental health. However, the rural elderly in the middle and west regions depend more on financial support from their children, the beneficial effects of pension income may be muted by the crowding-out effect on transfers from children [68]. Thus, the beneficial effects of pension income on mental wellbeing in the middle and west regions are not statistically significant.

### 5.3. Heterogeneous Effects of the NRSPI Pensions on Mental Health Outcomes

According to the WHO [12], depression is more common among females than males. We divide the sample into two groups according to the gender of the main respondents, to examine heterogeneous effects of the NRSPI pensions on mental health by gender. The results are reported in the first two columns of Table 7.

For males, the impact of pension income on CES-D score is negative, on rate of depressive symptoms or high depressive symptoms both are positive, and the coefficients are all insignificant. For females, the CES-D score, and the rates of depressive symptoms and high depressive symptoms are 0.387 points, 3.8, and 1.1 percentage points lower among pensioners respectively, and the coefficient of depressive symptoms is significant at the statistical level of 10%. Thus, the NRSPI pensions produce differential mental health effects by gender: pension income has no beneficial effects on mental health of older males, but improves that of older females. This finding is observed because older males and females may respond to income shocks differently [69]. Pension income from the NRSPI program may increase the health risk behavior of older males, such as smoking and drinking, whereas it may reduce labor supply and increase participation in social activities of older females and thus improve their mental health.

According to living arrangement information in 2011, we divided the sample into two categories, living independently and living in an extended household, to examine the effects of pension income on mental health by living arrangement dimension. The results are reported in the third and fourth columns of Table 7.

For the rural elderly living independently, the CES-D score, and the rates of depressive symptoms and high depressive symptoms are 0.384 points, 0.9, and 1.4 percentage points lower among the pensioners respectively, and the coefficients of the CES-D score are significant at the statistical level of 10%. For the rural elderly living in an extended household, the results show that the effects of pension income on CES-D score and rate of high depressive symptoms are both positive, on rate of depressive symptoms negative, but all are insignificant. Therefore, the NRSPI pension improves the mental health of elderly persons living independently, whereas it has no beneficial effects on those living in an extended household. This finding is observed because more than 50% of rural elderly live with their children or others, the pension income they receive may work in part to protect nutritional status and reduce psychosocial stress of their family members [31,33].

We divided the sample into two groups based on household net income in 2011, to examine the impact of pension income on mental health by household income dimension. The results are reported in the last two columns of Table 7.

The results show that the effect of pension income on CES-D score is positive, on rates of depressive symptoms and high depressive symptoms both are negative for the higher income group, and the coefficients are all insignificant. For the lower income group, the CES-D score, and rates of depressive symptoms and high depressive symptoms are 0.439 points, 0.8, and 0.3 percentage points lower among pensioners respectively, and the coefficient of the CES-D score is significant at the statistical level of 10%. Thus, the NRSPI pensions bring about differential mental health effects by household income, and those among the lower income group benefit from pension income. This finding is explainable because pension income is more important for the relatively poor elderly. Pension income helps improve their life quality and reduce their psychosocial stress.

### 5.4. Validity of the Instrument Variable

First, the timing of the NRSPI implementation at the county level is randomly determined by central government and is uncorrelated with pre-program mental health status. The results of the first-stage regression in Table 4, Table 6 and Table 7 show that the effects of the NRSPI implementation on pension income are all positive and significant at the statistical level of 1%, and the F test statistics far exceed the usual threshold value 10 for weak-strong IV. Thus, implementation of the NRSPI program in sample areas is a strong instrument variable for pension income.

Second, the NRSPI program complies with the current *hukou* policy, rural older adults who are eligible to receive pension benefits must have a local *hukou.* A Chinese person can change his or her *hukou* status through two channels, that is, going to college or marriage. Therefore, it is very difficult for elderly persons in rural areas to migrate and change their *hukou* locations from a county without the NRSPI program to one with the program to obtain pension benefits.

Third, pension coverage is associated with entitlements to different forms of health insurance in some LMICs, which makes it difficult to separate out the effects of pension income and health insurance [14]. However, in rural China, entitlement to the NRSPI program is not bundled in with that of the New Cooperative Medical Scheme (NCMS). The NCMS was first implemented in 2003, and covered all rural counties in 2008. In addition, there is no evidence that the NRSPI county had better mental health than the non-NRSPI county before the introduction of the NRPSI program [33].

## 6. Conclusions

The NRSPI program provides a prevalent but modest income security for the rural elderly in China. This paper first established a theoretical framework for the impact of the NRSPI pensions on mental wellbeing based on the agricultural household model and the health production model, and described plausible pathways from pension income to mental wellbeing. It then used CHARLS survey data of 2011 and 2013 to estimate the causal effect of the NRSPI pensions on mental health, and discussed the heterogeneous effects of pension income. We addressed the endogeneity of pension income by applying the fixed effect model with instrument variable.

Several important findings emerge from this study. First, the NRSPI pensions bring about small reductions in depression of the rural elderly by significantly improving their responses to “I felt depressed”. Second, the NRSPI pensions produce differential mental health effects by region: it slightly reduces depression of the rural elderly in the middle and west regions, whereas it has no beneficial effects on relieving that of those in the east region. Third, heterogeneous effects of the NRSPI pensions by gender, living arrangements, and household net income indicate that the beneficial effects of pension income are more pronounced among older females, elderly persons living independently, and those among the lower income group.

Our findings have critical policy implications. First, because the beneficial effects of the NRSPI pensions on mental health of the rural elderly are very limited, government should increase investment in mental illness prevention and treatment in rural areas, provide suitable mental health services, and help the rural elderly relieve depression. Second, because pension income has some beneficial effects on the mental health of pensioners with a lower income, further improvements and reforms to the NRSPI system should give more subsidies to elderly persons with poor economic conditions, especially those in the middle and west regions, to help raise income and improve mental wellbeing.

## Figures and Tables

**Table 1:** New Rural Society Pension Insurance (NRSPI) pension receipt, CES-D score and depressive symptoms.

	Pensioners			Non-Pensioners		
	Obs	Mean	Std	Obs	Mean	Std
CES-D score	1103	8.976	6.198	1621	9.727	6.449
Depressive symptoms	1103	0.510	0.500	1621	0.573	0.495
High depressive symptoms	1103	0.391	0.488	1621	0.455	0.498

**Table 2:** NRSPI pension receipt, CES-D score and depressive symptoms by region.

	East Region		Middle and West Regions	
	Pensioners	Non-Pensioners	Pensioners	Non-Pensioners
Sample size	217	479	835	1067
Mean of CES-D score	7.857	8.545	9.284	10.266
	[5.223]	[6.343]	[6.385]	[6.392]
Rate of depressive symptoms	0.438	0.480	0.533	0.616
	[0.497]	[0.500]	[0.499]	[0.487]
Rate of high depressive symptoms	0.359	0.365	0.402	0.497
	[0.481]	[0.482]	[0.491]	[0.500]

Standard deviations are in square parenthesis.

**Table 3:** Variable definition and descriptive statistics.

	Pensioners			Non-pensioners		
	Obs	Mean	Std	Obs	Mean	Std
Natural logarithm of NRSPI pension income (Pension)	1103	6.636	0.662	1621	0.000	0.000
Age of the main respondents (Age)	1103	68.927	5.862	1621	67.984	6.149
Marital status (Married)	1103	0.713	0.453	1621	0.719	0.449
Number of household members (Family)	1103	3.149	1.835	1621	3.355	1.976
Number of children alive (Children)	1103	3.694	1.626	1621	3.592	1.617
Have health insurance (Insurance)	1103	0.976	0.155	1621	0.943	0.233
Number of diagnosed chronic diseases (Chronic)	1103	1.310	1.298	1621	1.442	1.359
Cultivated land area operated (Land)	1103	4.728	12.804	1621	4.234	10.807
Natural logarithm of financial assets (Liquidity)	1103	5.787	3.007	1621	5.293	3.205

**Table 4:** Impact of the NRSPI pensions on mental health outcomes.

	FE Estimates		FE-IV Estimates	
	Coefficient	Standard Error	Coefficient	Standard Error
The second-stage regression:				
CES-D score	−0.035	0.042	−0.207	0.158
Depressive symptoms	−0.004	0.004	−0.013	0.014
High depressive symptoms	−0.002	0.004	−0.001	0.013
The first-stage regression:				
Impact of IV on pension income			2.491 ***	0.346
F test statistic of IV			84.08	
Number of observations	2724		2724	

*** indicates the significance level of 1%. Age, quadratic age, marital status, number of children alive, family size, health insurance status, number of diagnosed chronic diseases, cultivated land area operated and natural logarithm of the balance of financial assets are controlled.

**Table 5:** Impact of the NRSPI pensions on mental health outcomes by each item.

	FE Estimates		FE-IV Estimates	
	Coefficient	Standard Error	Coefficient	Standard Error
I was bothered by things that don’t usually bother me	−0.003	0.010	−0.024	0.031
I had trouble keeping my mind on what I was doing	−0.010	0.010	−0.014	0.033
I felt depressed	−0.001	0.009	−0.069 **	0.031
I felt everything I did was an effort	1.151 × 10^−4^	0.010	−0.001	0.034
I felt hopeful about the future	0.019	0.012	0.002	0.041
I felt fearful	−0.012 *	0.006	−0.010	0.023
My sleep was restless	−0.007	0.009	0.019	0.028
I was happy	0.014	0.011	−0.035	0.037
I felt lonely	−0.024 ***	0.008	−0.043	0.028
I could not get ‘going’	−0.012	0.007	−0.033	0.024

***, **, and * indicate the significance levels of 10%, 5%, and 1% respectively. Age, quadratic age, marital status, number of children alive, family size, health insurance status, number of diagnosed chronic diseases, cultivated land area operated and natural logarithm of the balance of financial assets are controlled.

**Table 6:** Impact of the NRSPI pensions on mental health outcomes by region.

	East Region		Middle and West Regions	
	Coefficient	Standard Error	Coefficient	Standard Error
The second-stage regression:				
CES-D score	0.011	0.226	−0.233	0.209
Depressive symptoms	0.005	0.021	−0.019	0.016
High depressive symptoms	0.016	0.016	−0.004	0.017
The first-stage regression:				
Impact of IV on pension income	3.297 ***	0.526	2.260 ***	0.440
F test statistic of IV	21.16		62.38	
Number of observations	696		1902	

*** indicates the significance level of 1%. Age, quadratic age, marital status, number of children alive, family size, health insurance status, number of diagnosed chronic diseases, cultivated land area operated and natural logarithm of the balance of financial assets are controlled.

**Table 7:** Heterogeneous effects of the NRSPI pensions on mental health outcomes.

	Male	Female	Living Independently	Co-residence	Above Median HH Income	Below Median HH Income
The second-stage regression:						
CES-D score	−0.031	−0.387	−0.384 *	0.019	0.029	−0.439 *
	[0.190]	[0.246]	[0.196]	[0.266]	[0.204]	[0.255]
Depressive symptoms	0.009	−0.038 *	−0.009	−0.017	−0.019	−0.008
	[0.018]	[0.020]	[0.016]	[0.022]	[0.018]	[0.020]
High depressive symptoms	0.007	−0.011	−0.014	0.016	−4.549 × 10^−4^	−0.003
	[0.018]	[0.018]	[0.016]	[0.022]	[0.018]	[0.018]
The first-stage regression:						
Impact of IV on pension income	2.609 ***	2.362 ***	2.971 ***	2.093 ***	2.486 ***	2.519 ***
	[0.400]	[0.443]	[0.436]	[0.392]	[0.367]	[0.439]
F test statistic of IV	63.34	57.99	69.27	52.67	61.03	60.98
Number of observations	1404	1320	1210	1514	1352	1372

*** and * indicate the significance levels of 10% and 1% respectively. Standard errors are in square parenthesis. Age, quadratic age, marital status, number of children alive, family size, health insurance status, number of diagnosed chronic diseases, cultivated land area operated and natural logarithm of the balance of financial assets are controlled.

## Data Availability

Data was obtained from the China Health and Retirement Longitudinal Study (CHARLS) dataset and are available at http://charls.pku.edu.cn/pages/data/111/zh-cn.html (accessed on 5 November 2020) with permission.

## Supplementary Materials

The following are available online at https://www.mdpi.com/1660-4601/18/5/2391/s1, Table S1: CES-D 10 questions.

## References

[1] United Nations (2019). World Population Prospects 2019. https://www.un.org/development/desa/pd/node/1114.

[2] Bongaarts J. (2009). Human population growth and the demographic transition. Philos. Trans. R. Soc. Lond. B. Biol. Sci..

[3] Suzman R., Beard J.R., Boerma T., Chatterji S. (2015). Health in an aging world—What do we know?. Lancet.

[4] Jung J., Tran C. (2012). The extension of social security coverage in developing countries. J. Dev. Econ..

[5] National Bureau of Statistics (2011). The 2010 Population Census of the People’s Republic of China. http://www.stats.gov.cn/tjsj/pcsj/rkpc/6rp/indexch.htm.

[6] National Bureau of Statistics (2020). Statistical Communiqué of the People’s Republic of China on the 2019 National Economic and Social Development. http://www.stats.gov.cn/tjsj/zxfb/202002/t20200228_1728913.html.

[7] United Nations World Population Aging 2009. https://www.un.org/en/development/desa/publications/world-population-ageing-2009.html.

[8] Byers A.L., Covinsky K.E., Barnes D.E., Yaffe K. (2012). Dysthymia and depression increase risk of dementia and mortality among older veterans. Am. J. Geriatr. Psychiatry.

[9] Collins P.Y., Patel V., Joestl S.S., March D., Insel T.R., Daar A.S., Bordin I.A., Costello E.J., Durkin M., Fairburn C. (2011). Grand challenges in global mental health. Nature.

[10] Byers A.L., Yaffe K., Covinsky K.E., Friedman M.B., Bruce M.L. (2010). High occurrence of mood and anxiety disorders among older adults: The National Comorbidity Survey Replication. Arch. Gen. Psychiatry.

[11] World Health Organization (2014). Mental Health Action Plan: 2013–2020. https://www.who.int/initiatives/mental-health-action-plan-2013-2030.

[12] World Health Organization (2017). Depression and Other Common Mental Disorders: Global Health Estimates. https://www.who.int/publications/i/item/depression-global-health-estimates.

[13] Chen X., Wang T., Busch S.H. (2019). Does money relieve depression? Evidence from social pension expansion in China. Soc. Sci. Med..

[14] LIoyd-Sherlock P., Agrawal S. (2014). Pensions and the health of older people in South Africa: Is there an effect?. J. Dev. Stud..

[15] Cheng L., Liu H., Zhang Y., Shen K., Zeng Y. (2015). The impact of health insurance on health outcomes and spending of the elderly: Evidence from China’s New Cooperative Medical Scheme. Health Econ..

[16] Adler N.E., Boyce W.T., Chesney M.A., Folkman S., Syme S.L. (1993). Socioeconomic inequalities in health: No easy solution. J. Am. Med. Assoc..

[17] Feinstein J.S. (1993). The relationship between socioeconomic status and health: A review of the literature. Milbank Q..

[18] Deaton A., Paxson C. (1998). Aging and inequality in income and health. Am. Econ. Rev..

[19] Smith J. (1999). Healthy bodies and thick wallets: The dual relation between health and economic status. J. Econ. Perspect..

[20] Ettner S.L. (1996). New evidence on the relationship between income and health. J. Health. Econ..

[21] Meer J., Miller D.L., Rosen H.S. (2003). Exploring the health–wealth nexus. J. Health Econ..

[22] Frijters P., Haisken-DeNew J.P., Shields M.A. (2005). The causal effect of income on health: Evidence from German reunification. J. Health Econ..

[23] Gardner J., Oswald A.J. (2007). Money and mental wellbeing: A longitudinal study of medium-sized lottery wins. J. Health Econ..

[24] Apouey B., Clark A.E. (2015). Winning big but feeling no better? The effect of lottery prizes on physical and mental health. Health Econ..

[25] Lindahl M. (2005). Estimating the effect of income on health and mortality using lottery prizes as an exogenous source of variation in income. J. Hum. Resour..

[26] Stillman S., McKenzie D., Gibson J. (2009). Migration and mental health: Evidence from a natural experiment. J. Health Econ..

[27] Milligan K., Stabile M. (2011). Do child tax benefits affect the well-being of children? Evidence from Canadian child benefit expansions. Am. Econ. J. Econ. Policy.

[28] Stowasser T., Heiss F., McFadden D., Winter J., Wise D.A. (2012). “Healthy, Wealthy and Wise?” Revisited: An analysis of the causal pathways from socioeconomic status to health. Investigations in the Economics of Aging.

[29] Kim J., Durden E. (2007). Socioeconomic status and age trajectories of health. Soc. Sci. Med..

[30] Golberstein E. (2015). The effects of income on mental health: Evidence from the social security notch. J. Ment. Health Policy Econ..

[31] Case A., Wise D.A. (2004). Does money protect health status? Evidence from South African pensions. Perspectives on the Economics of Aging.

[32] Galiani S., Gertler P., Bando R. (2016). Non-contributory pensions. Labour Econ..

[33] Cheng L., Liu H., Zhang Y., Zhao Z. (2018). The health implications of social pensions: Evidence from China’s New Rural Pension Scheme. J. Comp. Econ..

[34] Grossman M. (1972). On the concept of health capital and the demand for health. J. Political Econ..

[35] Lei X., Sun X., Strauss J., Zhang P., Zhao Y. (2014). Depressive symptoms and SES among the mid-aged and elderly in China: Evidence from the China Health and Retirement Longitudinal Study national baseline. Soc. Sci. Med..

[36] Singh I., Squire L., Stranss J. (1986). Agricultural Household Models: Extension, Applications and Policy.

[37] Cheng J. (2014). The effect of old-age security on labor supply. Econ. Res. J..

[38] Lemieux T., Milligan K. (2008). Incentive effects of social assistance: A regression discontinuity approach. J Econometrics..

[39] Shen C., Williamson J.B. (2010). China’s New Rural Pension Scheme: Can it be improved?. Int. J. Sociol. Soc. Policy..

[40] Chen X. (2017). Old age pension and intergenerational living arrangements: A regression discontinuity design. Rev. Econ. Househ..

[41] State Council of China (2009). Guidelines for the Pilot Implementation of the New Rural Pension Scheme. http://www.gov.cn/zhengce/content/2009-09/04/content_7280.htm.

[42] State Council of China (2014). Opinions of the State Council on Establishing a Unified Basic Pension Insurance System for Urban and Rural Residents. http://www.gov.cn/zhengce/content/2014-02/26/content_8656.htm.

[43] Liu Z. (2019). Impact of the Basic Pension Program on labor supply and retirement decisions: An empirical analysis based on the China Health and Retirement Longitudinal Study. Econ. Res. J..

[44] China Health and Retirement Longitudinal Study. http://charls.pku.edu.cn/pages/data/111/zh-cn.html.

[45] Radloff L.S. (1977). The CES-D scale: A self-report depression scale for research in the general population. Appl. Psychol. Meas..

[46] Bailly D., Beuscart R., Collinet C., Alexandre J.Y., Parquet P.J. (1992). Sex differences in the manifestations of depression in young people. A study of French high school student part I: Prevalence and clinical data. Eur. Child. Adolesc. Psychiatry.

[47] Andreasen E.M., Malmgren J.A., Carter W.B., Patrick D.L. (1994). Screening for depression in well older adults: Evaluation of a short form of the CES-D. Am. J. Prev. Med..

[48] Fernald L.G., Gunnar M.R. (2009). Poverty-alleviation program participation and salivary cortisol in very low-income children. Soc. Sci. Med..

[49] Barid S., de Hoop J., Özler B. (2013). Income shocks and adolescent mental health. J. Hum. Resour..

[50] Collins A.L., Goldman N. (2008). Perceived social position and health in older adults in Taiwan. Soc. Sci. Med..

[51] Lee M.C., Huang N. (2015). Changes in self-perceived economic satisfaction and mortality at old ages: Evidence from a survey of middle-aged and elderly adults in Taiwan. Soc. Sci. Med..

[52] Xiang Y.T., Yu X., Sartorius N., Ungvari G.S., Chiu H.F.K. (2012). Mental health in China: Challenges and progress. Lancet.

[53] Young D.K., Ng P.Y. (2016). The prevalence and predictors of self-stigma of individuals with mental health illness in two Chinese cities. Int. J. Soc. Psychiatry.

[54] Wong D.F.K., Xuesong H., Poon A., Lam A.Y.K. (2012). Depression literacy among Chinese in Shanghai, China: A comparison with Chinese-speaking Australians in Melbourne and Chinese in Hong Kong. Soc. Psych. Psych. Epid..

[55] The Economist (2017). China Wakes Up to Its Mental-Health Problems. http://refhub.elsevier.com/S0277-9536(18)30677-4/sref57.

[56] Chen X., Eggleston K., Sun A. (2017). The impact of social pension on intergenerational relationships: Comparative evidence from China. J. Econ. Aging.

[57] Woo J., Lynn H., Lau W.Y., Leung J., Lau E., Wong S.Y.S., Kwok T. (2006). Nutrient intake and psychological health in an elderly Chinese population. Int. J. Geriatr. Psych..

[58] Zhang Y., Liu Z., Cheng L. (2016). Does China’s New Rural Pension Scheme improve the life quality of the rural elderly?. China Econ. Q..

[59] Shu L. (2018). The effect of the New Rural Social Pension Insurance Program on the retirement and labor supply decision in China. J. Econ. Ageing.

[60] Chen X. (2016). Old-age pension and extended families: How is adult children’s internal migration affected?. Contemp. Econ. Policy.

[61] Payne L., Mowen A.J., Montoro-Rodriguez J. (2006). The role of leisure style in maintaining the health of older adults with arthritis. J. Leis. Res..

[62] Ning M., Gong J., Zheng X., Zhuang J. (2016). Does New Rural Pension Scheme decrease elderly labor supply? Evidence from CHARLS. China Econ. Rev..

[63] Chen H., Zeng Y. (2013). Who benefits more from the New Rural Society Endowment Insurance Program in China: Elderly or their adult children?. Econ. Res. J..

[64] Ma G., Zhou G. (2014). The impacts of New Rural Pension Program on household saving: Evidence from CFPS. Econ. Res. J..

[65] Wooldridge J.M. (2002). Econometric Analysis of Cross Section and Panel Data.

[66] Stock J.H., Wright J.H., Yogo M. (2002). A survey of weak instruments and weak identification in GMM. J. Bus. Econ. Stat..

[67] Baicker K., Taubman S.L., Allen H.L., Bernstein M., Gruber J.H., Newhouse J.P., Schneider E.C., Wright B.J., Zaslavsky A.M., Finkelstein A.N. (2013). The Oregon experiment-effects of Medicaid on clinical outcomes. N. Engl. J. Med..

[68] Wang F., Li R. (2016). Regional difference analysis on the substitutability of the New Rural Social Pension Insurance and family support. Ins. Stu..

[69] Jensen R.T., Richter K. (2004). The health implications of social security failure: Evidence from the Russian pension crisis. J. Public Econ..

