# Supplementary material for: Do Public Pensions Improve Mental Wellbeing? Evidence from the New Rural Society Pension Insurance Program
Source: Int J Environ Res Public Health. 2021 Mar 1;18(5):2391. doi: 10.3390/ijerph18052391 (PMC7967743; doi:10.3390/ijerph18052391)
Supplement: Supplementary file 1 [file ijerph-18-02391-s001.pdf]

International Journal of
Environmental Research and Public Health

MDPI

Table S1 CES-D 10 questions

DC009 I was bothered by things that don't usually bother me
DC010 I had trouble keeping my mind on what I was doing
DC011 I felt depressed
DC012 I felt everything I did was an effort
DC013 I felt hopeful about the future
DC014 I felt fearful
DC015 My sleep was restless
DC016 I was happy
DC017 I felt lonely
DC 018 I could not get "going"
